# A comparative study of human T-cell lymphotropic virus-associated myelopathy in HIV-positive and HIV-negative patients in KwaZulu-Natal

**DOI:** 10.4102/sajhivmed.v18i1.746

**Published:** 2017-12-06

**Authors:** Hoosain F. Paruk, Ahmed I. Bhigjee, Suzaan Marais

**Affiliations:** 1 Department of Neurology, Nelson R Mandela School of Medicine, University of KwaZulu-Natal, South Africa

## Abstract

**Background:**

KwaZulu-Natal is an endemic area for HIV and human T-cell lymphotropic virus (HTLV) infection. The main neurological manifestation of HTLV is HTLV-associated myelopathy/tropical spastic paraparesis (HAM/TSP). The effect of HIV co-infection in patients with HAM/TSP is not well documented.

**Aims:**

To determine the prevalence of HIV seropositivity in patients with HAM/TSP and compare the clinical, laboratory and radiological features of patients mono-infected with HTLV and those dually infected with HTLV and HIV.

**Methods:**

Adult patients referred to the Neurology Department at Inkosi Albert Luthuli Central Hospital in KwaZulu-Natal, South Africa, for the period 01 January 2004 to 31 December 2015 with a positive HTLV serology were identified from the National Health Laboratory Service database. A retrospective chart review was conducted to identify all patients who had a diagnosis of HAM/TSP and to record their HIV status. Clinical, laboratory and radiological data were compared for HIV-positive and HIV-negative patients.

**Results:**

A total of 52 patients with HAM/TSP were identified. HIV results were available in 44 patients of whom 23 (52%) patients were HIV co-infected. Patients who were HIV-positive had a younger age of presentation compared to HIV-negative patients (median: 31 vs 50 years, *p* = 0.002). HIV-positive patients had a median duration of symptoms at presentation of 12 months compared to 16 months for HIV-negative patients, but the difference did not reach statistical significance (*p* = 0.082). The CD4 cell counts of HIV-positive patients were well preserved with a median count of 781 cells/µL.

**Conclusions:**

HIV co-infection is commonly seen in the setting of HAM/TSP in KwaZulu-Natal. An interaction between the viruses may accelerate the development of HAM/TSP, leading to a younger age of presentation. Co-infection may have treatment implications because of CD4 counts being preserved in these patients.

## Introduction

The human T-cell lymphotropic virus (HTLV) is a retrovirus that is structurally similar to human immunodeficiency virus (HIV).^1^ Two HTLV types (HTLV-1 and HTLV-2) were identified to cause disease in humans prior to the identification of HIV. HTLV is spread in a manner similar to HIV, vertically and horizontally. The main form of spread of HTLV-1 is through sexual transmission and sharing of contaminated needles for HTLV-2.^2^

Human T-cell lymphotropic virus-1 is found in endemic clusters in various regions of the world, including KwaZulu-Natal. A population-based study performed in KwaZulu-Natal in 1992 on 1018 individuals showed a prevalence rate of 2.6% (95% confidence interval (CI) 1.62–3.58).^3^ In this study, only one case of HTLV-2 was identified. The HIV-positive rate in the same cohort was 3.5% (95% CI 2.4–4.68) and the relative risk of co-infection in the 15–24 year age group was 1.16 (95% CI 1.08–1.24). HTLV-1 is associated with the development of neurological disease, the most important of which is HTLV-1-associated myelopathy/tropical spastic paraparesis (HAM/TSP). HIV leads to neurological complications in more than 40% of infected patients, including vacuolar myelopathy, a condition that clinically resembles HAM/TSP.^4^

The occurrence of co-infection is not unexpected in areas endemic for both viruses and considering their common modes of transmission. One of the earliest reports of retroviral co-infection found that 7% of patients with AIDS were seropositive for HTLV.^5^ Of the 90 patients who were identified with HAM/TSP during the initial epidemiological study in KwaZulu-Natal in 1992, six (6.6%) were co-infected with HIV.^3^ In a more recent retrospective analysis,^6^ 24% of samples tested positive for HTLV. This Limpopo analysis included 170 HIV-positive samples collected during 2007 and 2008.

The consequences of co-infection on viral replication, pathogenicity and susceptibility to antiretroviral therapy (ART) are critical issues, which require investigation. Early studies of retroviral co-infection, in particular with HTLV-1, performed before the era of ART suggested that HTLV seropositivity accelerated the progression of HIV to AIDS.^7,8,9,10^ The lifetime risk of developing HAM/TSP in HTLV-1 seropositive individuals is estimated to be around 3% – 5%, but a report of HAM/TSP developing in three out of four patients with co-infection in the pre-ART era was the first to suggest that myelopathy risk is increased with co-infection.^11,12^ Ascertaining the cause of myelopathy in such patients can be challenging because of the similarities in clinical presentation. Whilst the CD4 cell count is used as a marker for immunosuppression in HIV infection, HTLV has been shown to have lymphoproliferative effects.^13^ This complicated management in resource-constrained settings when the decision to commence ART in HIV-positive patients was based on the peripheral blood CD4 count. A direct comparison of HAM/TSP in HIV-positive and negative patients has only been reported in one retrospective study that examined the records of nine dually infected patients and 11 HTLV mono-infected patients with HAM/TSP.^14^ The study suggested that patients with HAM/TSP present earlier in dually infected patients. ART has not been shown to be effective in the treatment of HAM/TSP; also the response of the HIV viral load in the setting of dually infected patients is not well documented.^15^

Thus, despite the potential clinical significance, the possibility of co-infection is often overlooked by clinicians as HTLV is not routinely tested in HIV outpatient settings. The aim of this hospital-based study was to determine the prevalence of HIV seropositivity in patients with HAM/TSP and compare the clinical, laboratory and radiological features of patients mono-infected with HTLV and those dually infected with HTLV and HIV.

## Method

### Patients

The Inkosi Albert Luthuli Central Hospital (IALCH) is one of the two neurology referral centres in the province of KwaZulu-Natal which has an adult population of 7.5 million individuals over the age of 15 years. This hospital is the referral centre for the northern and southern parts of KwaZulu-Natal, an area that is endemic for HTLV infection.

A record of all seropositive HTLV results for patients referred to the Neurology Department at IALCH for the period 01 January 2004 to 31 December 2015 was obtained from the National Health Laboratory Services (NHLS) electronic database. The NHLS uses an ARCHITECT rHTLV-I/II ELISA test to detect HTLV seropositivity. This method has a sensitivity of 100% (95% CI 99.10–100) and specificity >99.95% (95% CI 99.84–99.99). It does not distinguish between HTLV-1 and HTLV-2. It is assumed that most, if not all, of the cases represent HTLV-1 infection considering that HTLV-2 is an infrequent cause of myelopathy and that only one case of HTLV-2 was detected in a previous epidemiological study in KwaZulu-Natal.^3^ A chart review was performed on all patients with a positive HTLV serology result to identify patients who had a diagnosis of HAM/TSP. The diagnosis was based on a combination of clinical, radiological and laboratory findings. Investigations to exclude an alternative cause for myelopathy that were performed included the following: (1) blood tests including Vitamin B12 levels and syphilis serology, (2) cerebrospinal fluid (CSF) analysis, including biochemistry, cytology, microbiology (including microscopy and culture for fungi, pyogenic bacteria and *Mycobacterium tuberculosis*), syphilis serology and Cryptococcus latex agglutination titre and (3) Magnetic resonance imaging (MRI) of the spine. Patients without myelopathy or with myelopathy because of other causes were excluded. HIV status of the patients was also ascertained where available. The clinical, laboratory and radiological features of these patients were extracted from the patient records using a structured data sheet. MRI spine was reported by radiologists and subsequently reviewed by the study team (all qualified neurologists).

### Data analysis

The data collected were analysed using Prism Version 6. Descriptive statistics such as frequencies, percentages, medians and interquartile ranges were used to summarise results. Continuous and categorical variables were analysed using Mann–Whitney U test and Fischer’s exact test, respectively. A *p*-value of <0.05 was considered statistically significant.

## Results

A total of 121 patients had a positive HTLV serology result during the study period; 52 patients had a diagnosis of HAM/TSP and were included in the study. HIV results were available for 44 of these patients. Twenty-three (52%) patients were HIV-positive and the rest were HIV-negative.

### Demographic characteristics

Demographic and clinical features are summarised in Table 1. Patients who were HIV-positive had younger age of presentation compared to HIV-negative patients (median age: 31 vs. 50, *p* = 0.002). There were proportionately more females in both groups, with 17 (67%) and 14 (67%) in the HIV-positive and HIV-negative groups, respectively.

**TABLE 1:** Demographic and clinical features of human T-cell lymphotropic virus-associated myelopathy/tropical spastic paraparesis patients.

Variables	All (*n* = 52†)		HIV + (*n* = 23)		HIV − (*n* = 21)		HIV + vs. HIV – (*p*)
	Median/n	IQR/ %	Median/n	IQR/ %	Median/n	IQR/ %	
**Demographics**							
Age (years)	41	32–53	37	30–44	50	40–56	0.002
Gender (female)	35	67.3	17	73.9	14	66.6	0.74
**Clinical features**							
Duration of symptoms (months)	12	6–48	10	4–24	16	9–50	0.082
MRC power grade	4	2–4	4	2–4	3	1–4	0.54
Sensory impairment (yes)	37	74	14	60.9	19	90.5	0.037
Sphincter disturbance (yes)	26	52	9	39.1	15	71.4	0.040
Ambulant (yes)	26	52	14	60.9	9	30	0.37

† Clinical features data missing for two patients.

HIV+, HIV-positive; vs., versus; HIV−, HIV-negative; IQR, interquartile range; MRC, Medical Research Council; n, number; %, percentage.

### Clinical features

There was a trend for the duration of symptoms prior to diagnosis to be shorter in HIV-positive patients compared to HIV-negative patients, but this did not reach statistical significance (*p* = 0.082). There was no significant difference in the power grading or proportion of patients who remained ambulant in the two groups. However, there was a significantly increased frequency of sensory and sphincter disturbance in HIV-negative patients compared to HIV-positive patients (*p* = 0.037, *p* = 0.04). Four patients were receiving ART.

### Investigations

The CSF parameters are summarised in Table 2. There was a trend for HIV-positive patients to have higher protein and lower glucose values than the HIV-negative patients. In addition, polymorphonuclear cells were only detected in patients who were HIV-positive. CD4 cell counts were available in 16 of the HIV-positive patients (median: 781 cells/µL, range: 47 – 1993).

**TABLE 2:** Cerebrospinal fluid findings of human T-cell lymphotropic virus-associated myelopathy/tropical spastic paraparesis patients.

Variables	All patients (*n* = 33)		HIV + (*n* = 16)		HIV − (*n* = 15)		HIV + vs. HIV − (*p*)
	Median	IQR	Median	IQR	Median	IQR	
Protein (g/L)	0.45	0.31–0.74	0.63	0.37–1.01	0.41	0.27–0.54	0.099
Glucose (mmol/L)	3	2.6–3.23	2.9	2.45–3	3.2	2.7–3.4	0.023
Polymorphonuclear cells (cells/µL)	0	0–2.5	0	0–13	0	0–0	0.007
Lymphocytes (cells/µL)	1	0–8	2	0–10	0	0–8	0.58

HIV+, HIV-positive; vs., versus; HIV−, HIV-negative; IQR, interquartile range; CSF, cerebrospinal fluid.

Thoracic spinal cord atrophy, which was determined subjectively, was noted on MRI in eight out of 23 HIV-positive patients and seven (33.3%) out of the 19 HIV-negative patients who had imaging available. A hyperintense intramedullary signal was noted in six (25%) patients who were HIV-positive and none of those who were HIV-negative.

## Discussion

The occurrence of HAM/TSP in the setting of patients co-infected with HTLV and HIV was first described by Berger et al.^12^ Four of 241 HIV-positive patients were confirmed to be seropositive for HTLV during the course of a longitudinal survey of neurological complications of HIV. Three of the four patients, two HTLV-1 and one HTLV-2, had a progressive myelopathy that clinically resembled HAM/TSP.^16^ Subsequently, other studies have also showed increased rates of HAM/TSP in co-infected patients. A study in the pre-ART era by Harrison et al. did not diagnose HAM/TSP but described clinical evidence of myelopathy in 11 of 15 (73%) patients with HIV/HTLV-1 co-infection as compared to 10 of 62 (16%) patients with HIV mono-infection.^17^ Beille et al. screened 3600 HIV-positive patients at an outpatient clinic in the United States between 1993 and 2002 and confirmed 62 and 141 HIV/HTLV-1 and HIV/HTLV-2 co-infections, respectively. Four of the 62 (9.7%) HIV/HTLV-1-infected patients were diagnosed with HAM/TSP; two of the patients were receiving zidovudine monotherapy.^18^ ART does not appear to decrease the risk of HAM/TSP, as a study by Silva et al. reported myelopathy in 12 of 47 (25.5%) co-infected patients on ART.^19^ Casseb et al. diagnosed HAM/TSP in six out of 38 (18%) dually infected patients and found that HTLV-1 proviral loads were significantly higher in patients with HAM/TSP compared to asymptomatic co-infected patients (*p* = 0.012).^20^

Our study confirms a high frequency of HIV co-infection in patients with HAM/TSP in KwaZulu-Natal. Dual infection is associated with a younger age of clinical presentation and shorter duration of symptoms prior to presentation. We postulate that the presence of HIV infection in HTLV-infected individuals predisposes them to developing HAM/TSP and that the disease course is of a more accelerated nature. The possibility of a more aggressive disease would also explain our finding of spinal cord hyperintensity which was only observed in patients who were also HIV-infected. Atypical MRI findings such as hyperintensity and contrast enhancement have been associated with more acute progression in HAM/TSP.^21^ However, there was no significant difference in the power grading or proportion of patients who remained ambulant between the two groups, and the increased frequency of sensory and sphincter impairment in the HTLV mono-infected group is unexplained. The differences in the CSF findings of higher protein, lower glucose and increased white cells in HIV-positive patients are potentially a reflection of the chronicity of HIV infection and do not necessarily imply more aggressive disease.

The proportion of HAM/TSP patients found to be HIV-positive in this study is much higher than what was initially observed in KwaZulu-Natal province (52% vs. 6.6%). This is not unexpected given that the previous survey of HAM/TSP patients in the region was conducted in the early 1990s, a period which signalled the beginning of the HIV epidemic in South Africa. The finding is similar to that of a more recent South African study that investigated HIV co-infection in HAM/TSP patients in which nine of 21 (42.8%) patients with HAM/TSP were found to have concurrent HIV infection.^14^ Schutte et al. reported similar findings to ours with respect to age at presentation and duration of symptoms. However, a statistically significant difference was not demonstrated with regard to age at presentation, which might have been related to small sample size. CD4 counts in our cohort tended to be relatively preserved with the median count being 781 cells/µL, which differs from the study of Schutte et al. in which 56% of patients had counts less than 350 cells/µL. It should be noted that although preserved, these CD4 cells are functionally impaired and ART should be considered regardless of the CD4 counts in these patients.^22,23^

HIV leads to increased HTLV protein expression.^24,25^ These effects may be a possible explanation as to how HTLV is more readily able to overcome the immune response. Both HIV and HTLV infect endothelial cells and astrocytes, resulting in blood-brain barrier damage.^26^ The proportional contribution of each of the viruses to the development of myelopathy has been debated; however, an early pathological study of two patients with co-infection suggested that the myelopathy was because of HTLV as no evidence of HIV-associated vacuolar myelopathy was found.^27^ In a further post-mortem examination of a dually infected patient with HIV dementia and HAM/TSP, co-infection of a single-cell phenotype could not be demonstrated.^28^ HIV was found in macrophages or microglia, whilst HTLV was found in astrocytes, suggesting that mechanisms other than co-infection of the same cell play a role in the development of neurological disease in co-infected patients. Whilst HIV-associated neurocognitive disorder (HAND) has been well characterised, there is much less information regarding the effects of HTLV or retroviral co-infection on the brain and cognition.

Limitations of this study include the retrospective design, the inability to ascertain the sequence of viral infection and the unavailability of longitudinal data. We could not ascertain if ART offers any protection, nor could we determine whether ART alters the progression of disease. We also did not have any data regarding HIV and HTLV viral and proviral loads, respectively.

## Conclusion

To date this study is the largest that directly compares HAM/TSP in HIV-positive and HIV-negative patients and suggests that HTLV-1 and HIV have a synergistic effect with regard to the development of neurological complications. CD4 cell counts tend to be relatively well preserved in co-infected patients.

Further longitudinal studies of mono-infected and dually infected patients with monitoring of viral loads and clinical outcomes are required to enhance the understanding and impact of HIV/HTLV co-infection. Areas known to be endemic for both viruses provide a unique and ideal opportunity to perform community-based screening of asymptomatic patients. Ascertaining the sequence of infection, clinical manifestations, monitoring biomarkers of disease and response to ART are key issues requiring further investigation.
